# Development of Artocarpin-Loaded Chitosan Particles for Controlled Release and Inflammatory Application

**DOI:** 10.3390/polym18010008

**Published:** 2025-12-19

**Authors:** Piyapan Manklinniam, Phonchanok Reuk-ngam, Supavadee Boontha, Kunlathida Luangpraditkun, Sukunya Ross, Jarupa Viyoch, Atchariya Yosboonruang

**Affiliations:** 1 Division of Microbiology and Parasitology, School of Medical Sciences, University of Phayao, Phayao 56000, Thailand; 2 Laboratory of Organic Synthesis, Chulabhorn Research Institute, 54 Kamphaeng Phet 6, Talat Bang Khen, Lak Si, Bangkok 10210, Thailand; 3 School of Pharmaceutical Sciences, University of Phayao, Phayao 56000, Thailand; 4 Faculty of Pharmaceutical Sciences, Burapha University, Chonburi 20131, Thailand; 5 Center of Excellence in Biomaterials, Department of Chemistry, Faculty of Science, Naresuan University, Phitsanulok 65000, Thailand; 6 Department of Pharmaceutical Technology, Faculty of Pharmaceutical Sciences, Naresuan University, Phitsanulok 65000, Thailand

**Keywords:** artocarpin, chitosan microparticles, nitric oxide

## Abstract

Artocarpin, a flavonoid derived from *Artocarpus altilis*, has been reported to exhibit anti-inflammatory and geroprotective activities. In this study, artocarpin was isolated from *A. altilis* heartwood via maceration followed by chromatographic purification, yielding 0.435% of dried extract with a purity of approximately 81%, as confirmed by HPLC. To enhance the physicochemical stability and biological performance of artocarpin, a chitosan-based microparticle delivery system was developed using 0.1% chitosan cross-linked with 0.5% sodium tripolyphosphate (5:1 ratio). The optimized formulation achieved an encapsulation efficiency of 0.5 µg of artocarpin per mg of particles (loading content 0.05% *w*/*w*). Physicochemical analysis revealed that the particles possessed a predominantly spherical morphology with sizes ranging from 1 to 4 µm. The hydrodynamic diameter measured by DLS was approximately 3.3 µm, with a PDI of 0.79 ± 0.18 and a zeta potential of 12.8 mV, indicating acceptable dispersity and colloidal stability for a chitosan-based microparticle system. FTIR and XRD analyses verified successful incorporation of artocarpin into the chitosan matrix. In vitro release studies showed a biphasic pattern with an initial burst within 1–12 h followed by sustained release, reaching approximately 60% cumulative release. The anti-inflammatory activity of the formulations was evident through a dose-dependent reduction in nitric oxide production in LPS-stimulated RAW 264.7 macrophages. The artocarpin-loaded particles (CSPs/AE) suppressed NO levels by 34.33 to 73.19%, with statistically significant decreases at *p* < 0.05. These results highlight the potential of artocarpin-loaded chitosan microparticles as an effective anti-inflammatory delivery system with promising applicability for osteoarthritis management.

## 1. Introduction

Artocarpin, a flavonoid compound derived from *Artocarpus* spp. (family Moraceae), has demonstrated potent activities, including antibacterial, antitubercular, antioxidant, and cytotoxic effects [1]. It has also been shown to inhibit reactive oxygen species (ROS) production and modulate apoptosis-related pathways, suggesting its potential as a geroprotective and anti-inflammatory agent [2]. Artocarpin has been recognized as a potential anti-inflammatory agent due to its ability to downregulate key pro-inflammatory cytokines. In inflammatory conditions, the overproduction of cytokines such as tumor necrosis factor-α (TNF-α) is often accompanied by the upregulation of cyclooxygenase-2 (COX-2), which further amplifies the inflammatory response [3]. Notably, artocarpin has been shown to reduce the expression of interleukin-1β (IL-1β) [4] and interleukin-6 (IL-6) [5], both of which are central mediators in chronic inflammatory processes. In addition to its anti-inflammatory effects, artocarpin also inhibits the production of ROS and nitrite, as well as the activation and secretion of apoptosis-related molecules such as TNF-α [6]. Despite these promising pharmacological properties, its clinical application is hindered by poor water solubility and low bioavailability, highlighting the need for an optimized delivery system to enhance its therapeutic efficacy. However, there is still a lack of delivery systems specifically designed to protect artocarpin from degradation and provide sustained release. Chitosan is an appropriate carrier due to its biocompatibility, cationic structure, and ability to enhance encapsulation efficiency and controlled release. Incorporating artocarpin into a chitosan-based microparticle system therefore offers a rational strategy to improve its stability and therapeutic performance.

Chitosan, a natural polysaccharide obtained from chitin, has attracted considerable interest as a drug delivery material due to its biocompatibility, biodegradability, and low toxicity [7]. Its cationic nature enables strong interactions with negatively charged biomolecules, supporting controlled cross-linking and the formation of stable polymeric matrices. In addition to its safety profile, chitosan possesses inherent antimicrobial and anti-inflammatory activities, which enhance its suitability for therapeutic applications [8]. Owing to its adaptable chemical structure, chitosan can be processed into various delivery platforms such as nanoparticles, microparticles, and hydrogels, allowing modulation of drug release and improvement of the stability and solubility of poorly water-soluble compounds [9,10,11,12]. Chitosan-based microparticles, in particular, provide sustained release and effective protection of sensitive bioactive molecules, offering clear advantages for delivering compounds like artocarpin that require improved stability and controlled release [13,14].

Microparticles (MPs) have gained attention as advanced drug delivery platforms due to their high surface area, multi-compartment structure, and capability for controlled and targeted drug release. These features not only enhance drug bioavailability and therapeutic efficacy but also reduce systemic toxicity and improve patient compliance. MPs can be fabricated from both synthetic and natural polymers. While synthetic materials such as polylactic-co-glycolic acid (PLGA) and polycaprolactone (PCL) provide tunable degradation profiles and mechanical strength, natural polymers like alginate, gelatin, and chitosan offer superior biodegradability and biological functionality [15]. Nevertheless, many biopolymers suffer from mechanical limitations and low physicochemical stability, which can be addressed by incorporating structural modifiers such as fillers or nanofillers to enhance performance and broaden application potential [16].

This study aimed to (I) develop and optimize chitosan-based MPs (CSPs) for delivery of artocarpin extracted from *Artocarpus altilis* heartwood (CSPs/AE); (II) assess the physicochemical and biological properties of the biosynthesized CSPs/AE. This formulation combines natural bioactive compounds with drug delivery technology; this system offers a biocompatible approach for targeted osteoarthritis (OA) therapy and the management of inflammation-associated disorders.

## 2. Materials and Methods

### 2.1. Materials

Diethyl ether, hexane, ethyl acetate, and acetic acid were purchased from RCI Labscan (Bangkok, Thailand). Chitosan derived from crab shell, with low molecular weight, a deacetylation degree ≥ 75%, and a viscosity ≤ 15 mPa·s, was purchased from Maruzen chemicals (Tokyo, Japan). Sodium tripolyphosphate (TPP) was supplied by Thermo Scientific (Waltham, MA, USA). Dulbecco’s Modified Eagle Medium (DMEM), fetal bovine serum (FBS), trypsin-EDTA, and penicillin/streptomycin were purchased from Gibco Invitrogen™ (Grand Island, NY, USA). MTT (3-(4,5-Dimethylthiazol-2-yl)-2,5-Diphenyltetrazolium Bromide) was purchased from TCI (Tokyo, Japan), and silica gel from Merck (Darmstadt, Germany).

### 2.2. Artocarpin Extraction and Purification

The heartwood of *A. altilis* was chipped and dried at 45 °C for 3 days in a hot-air oven. These dried chips were ground into powder, then covered and macerated with diethyl ether at room temperature for two cycles (2 days per cycle), as described previously [17]. The ether extracts were pooled, filtered, and concentrated using a rotary evaporator. The extraction yield (%) was calculated using the equation:

Extraction yield (%) = (weight of dried extract/weight of dried heartwood powder) × 100

To obtain an extract enriched with artocarpin, column chromatography was employed. Silica gel No. 60 (column dimensions 3 × 50 cm) was used as the stationary phase, and the column was initially equilibrated with hexane. The crude extract (500 mg, dry weight) was dissolved in minimal solvent and loaded onto the column. Elution was performed using a gradient of ethyl acetate in hexane, and eluates were collected in 5 mL fractions. Each fraction was analyzed for flavonoid content using thin-layer chromatography (TLC) on silica gel 60 F254 with a mobile phase of hexane:ethyl acetate at 7:3 (*v*/*v*) and an approximate flow rate of 1 mL/min. Spots were visualized under UV light at 254 nm and 366 nm. Fractions exhibiting similar TLC profiles were pooled and subsequently concentrated using a rotary evaporator under reduced pressure. The final enriched artocarpin was stored at −20 °C for up to 3 months before further experimentation.

### 2.3. Artocarpin Characterization

Quantification of artocarpin in the extract was performed using isocratic high-performance liquid chromatography (HPLC), which consists of two low-pressure mixing pumps, a UV detector, a vacuum degasser, an automatic liquid sampler and a thermostatted column compartment (Dionex Softron, Germering, Germany; model: ultimate 3000). Separation was achieved using a C18 column (VertiSep™ UPS C18, 250 × 4.6 mm, 5 µm particle size). The mobile phase comprised acetonitrile and water in an 80:20 ratio (*v*/*v*), delivered at a constant flow rate of 1 mL/min, and maintained at a constant temperature of 30 °C. Purified artocarpin exhibited a maximum absorption wavelength (λmax) at 282 nm; therefore, UV detection was performed at 282 nm to ensure optimal sensitivity. Artocarpin content was quantified by comparing the integrated peak area obtained from the samples with a previously established calibration curve prepared using a standard of purified artocarpin [6]. All analyses were performed in triplicate using HPLC-grade solvents throughout.

### 2.4. Fabrication of Artocarpin Loaded Chitosan Microparticles

Chitosan microparticles were prepared using the ionotropic gelation method described by Mulia et al. [18]. First, chitosan was dissolved in a 1.0% *w*/*v* acetic acid solution to achieve a 0.1% *w*/*v* concentration. The pH of the solution was adjusted to 4.6 using a pH meter. TPP was dissolved in distilled water to obtain a 0.5% *w*/*v* concentration. The chitosan solution was magnetically stirred at 800 rpm for 24 h. The TPP solution was then added dropwise to the chitosan solution at a chitosan:TPP volume ratio of 5:1. The addition was performed at a controlled rate of approximately 0.5 mL/min, under continuous magnetic stirring at 1100 rpm for 24 h. Artocarpin extract was dissolved in 3% (*v*/*v*) aqueous propylene glycol and subsequently incorporated into the chitosan solution at a final concentration of 0.5 µg/mL, mixture was continuously stirred using a magnetic stirrer overnight at room temperature. The final suspension of CSPs/AE was centrifuged at 10,000 rpm for 25 min at 25 °C and the resulting pellet was collected. The microparticles were washed twice with deionized water and centrifuged again under the same conditions.

### 2.5. Determination of Particle Size, Polydispersity Index (PDI) and Zeta Potential and Stability Study

The physicochemical properties of particles, such as particle size, PDI, and zeta potential, were measured at 25 °C using a Zetasizer Nano (Malvern, UK) after appropriate dilution with deionized water. The experiments were conducted in triplicate, and all data were presented as the mean ± standard deviation (SD).

### 2.6. Encapsulation Efficiency (EE)

The encapsulation efficiency percentage (%EE) of artocarpin in the chitosan microparticles was determined by quantifying the amount of unencapsulated artocarpin in the supernatant using UV–Vis spectrophotometry at 320 nm, which corresponds to the maximum absorbance of the crude artocarpin extract in the UV–Vis spectrum. This wavelength differs from the 282 nm used in the HPLC method because HPLC detects purified artocarpin, whereas the UV–Vis measurement involves the extract, which contains additional chromophoric constituents that shift the absorbance maximum toward a higher wavelength. A formula was used to define the content of AE loaded in the microparticles: EE % was calculated using the following modified equation [19].

EE (%) = [C]*_i_* − [C]*_f_*/[C]*_i_* × 100

where [C]*_i_* is the Initial artocarpin concentration, µg/mL and [C]*_f_* is the concentration of unencapsulated artocarpin in the supernatant, µg/mL.

### 2.7. Cumulative Release

Artocarpin release from CSPs/AE was investigated in vitro using phosphate-buffered saline (PBS) of pH 7.4. A measured quantity (5 mg) of CSPs/AE was placed into 10 mL of PBS in a capped tube and inserted into a release medium preheated to 37 ± 0.5 °C with continuous stirring [20]. At predetermined time points (0, 1, 2, 4, 6, 8, 10, 12, 14, 16, 18, 24, and 48 h), 1 mL of the release medium was withdrawn and centrifuged at 10,000 rpm for 5 min to remove undissolved microparticles. The resulting supernatant was collected for analysis, and an equal volume of fresh, prewarmed PBS was immediately added to maintain a constant volume and preserve sink conditions. The amount of artocarpin released was investigated by measuring optical density at 320 nm. Finally, the amount of artocarpin retrieved at each time point was analyzed with a UV-Vis Spectrophotometer, and the cumulative release percentage (%CR) was calculated using the equation

CR% = Amount of drug released at time/Total amount of drug encapsulated × 100

### 2.8. Accelerated Stability Studies

Optimized CSPs and CSPs/AE were subjected to stability testing for three months as per ICH guidelines. The samples were stored at 40 ± 2 °C and 60% relative humidity (RH) in sealed containers and at 4 ± 3 °C for 90 days. The physicochemical stability of CSPs and CSPs/AE was assessed by monitoring changes in particle size (z-average); the formulations were periodically analyzed on days 0, 6, 30, and 90 for changes in particle size using dynamic light scattering (DLS). The results were recorded as the z-average diameter (mean particle size) and expressed as mean ± SD.

### 2.9. CSPs/AE Characterization

The dried biomass, following reduction, was subjected to Fourier transform infrared (FTIR) analysis to identify the chemical bonds and functional groups associated with the sample. This analysis was conducted in the range of 400–4000 cm^−1^ at a resolution of 4 cm^−1^ using an FTIR spectrometer (Nicolet 6700, Thermo Scientific (Waltham, MA, USA). X-ray diffraction (XRD) patterns of the synthesized particles were obtained via a Rigaku SmartLab^®^ X-ray diffractometer (Rigaku Corporation, Tokyo, Japan). The instrument employed Cu Kα radiation in a θ–2θ configuration and was operated at 40 kV and 30 mA. XRD analysis was used to confirm the crystalline structure of the particles.

The morphology and size of the microparticles were analyzed by scanning electron microscopy (SEM) (FEI Quanta 250, FEI Company, Hillsboro, OR, USA) with a magnification range of 5000 and an accelerating voltage 20–30 kV. For SEM images, the samples were sputter-coated with about 15 nm Au using a Polaron coater system. Transmission electron microscopy (TEM; FEI Tecnai G2 20, FEI Company, OR, USA) The samples were diluted at a ratio of 1:200 (*v*/*v*). A sample drop was then placed on a carbon-coated copper grid, followed by negative staining with a 1% aqueous solution of phosphotungstic acid after 15 min. The grid was air-dried thoroughly, and the samples were viewed on a TEM.

### 2.10. Cytotoxicity Activity

For cell culture, the macrophage cell line RAW 264.7 was cultured and maintained in Dulbecco’s modified Eagle’s medium (DMEM) high-glucose medium containing 5% fetal bovine serum (FBS) and 100 units/mL of penicillin-streptomycin. The cell cultures were incubated in a carbon dioxide incubator at 37 °C with 90% humidity and 5% CO_2_.

To determine cell cytotoxicity and evaluate the cytotoxicity of artocarpin, RAW 264.7 macrophage cells were seeded into 96-well plates at a density of 1.5 × 10^3^ cells/well and incubated for 18 h to allow cell attachment. Artocarpin was first dissolved in DMSO to prepare a stock solution and subsequently diluted with serum-free DMEM to obtain final concentrations of 0.1, 0.5, 1, 5, and 10 µg/mL. Cells were then treated with the prepared artocarpin solutions to assess cytotoxicity. After incubation, the culture medium was replaced with 100 µL of the artocarpin-containing media per well, and the cells were incubated for an additional 24 h. Subsequently, the MTT assay was performed to evaluate cell viability. Briefly, after the 24 h exposure period, the treated cells were gently washed twice with PBS to remove residual particles and treatment compounds. Then, 50 μL of MTT solution was added to each well, and the plates were incubated for 3 h to allow the formation of formazan crystals. The medium was carefully removed, and 100 μL of DMSO was added to each well to solubilize the formazan. The absorbance was measured at 570 nm using a microplate reader. Cell viability of CSPs/AE at the concentrations of 0.1, 1, 10, and 10 mg/mL was measured using the MTT assay as described above. The decrease in optical density caused by the compounds was used as a measurement of cell viability, compared to the control, which was considered 100% viable.

% Cell viability = Absorbance of the treatment/Absorbance of control × 100

### 2.11. Nitric Oxide (NO) Quantification Assay

The anti-inflammatory effects of the CSPs/AE as a nitric oxide (NO) regulator was investigated by measuring nitrite in the supernatants of cultured RAW 264.7 cells. The cells were seeded at 1 × 10^4^ cells/mL in 24-well culture plates and cultured for 18 h. After incubation, the culture medium was replaced with fresh serum-free medium containing lipopolysaccharide (LPS, 1 μg/mL) to induce inflammation. CSPs/AE were added at various concentrations (0.1, 1, 10, and 100 mg/mL) to the appropriate wells to assess their potential inhibitory effects on NO production. Cells treated with LPS alone and Polymyxin B served as the positive control, while untreated cells were used as the negative control (DMEM). The culture medium was mixed with an equal volume of Griess reagent and incubated at room temperature for 30 min. The concentrations of nitrite were measured by reading at 540 nm using a microplate reader, following a method modified from Önnheim et al. [21]. Nitrite production was determined by comparing the optical density with the standard curve obtained with sodium nitrite. All experiments were performed in triplicate. Nitrite concentrations were quantified using a sodium nitrite standard curve, and results were expressed as a percentage of NO inhibition relative to the LPS control, calculated using the following equation:

% NO inhibition = Absorbance of LPS − Absorbance of sample/Absorbance of LPS − Absorbance of DMEM × 100

### 2.12. Data Analysis

All experiments were performed in triplicate. Statistical analysis was conducted using one-way analysis of variance (ANOVA) followed by Tukey’s post hoc test in IBM SPSS Statistics 30.0. Data are presented as mean ± SD. Differences were considered statistically significant at *p* < 0.05.

## 3. Results and Discussion

### 3.1. Artocarpin Characterization

Extraction Yield: Dried heartwood of *A. altilis* (2000 g) was extracted with diethyl ether at room temperature for two cycles. After filtration and solvent removal, the crude extract yield was 8.7 g, corresponding to 0.435% of the dry weight, or approximately 0.0005 g per gram of dried heartwood.

**Figure 1:**
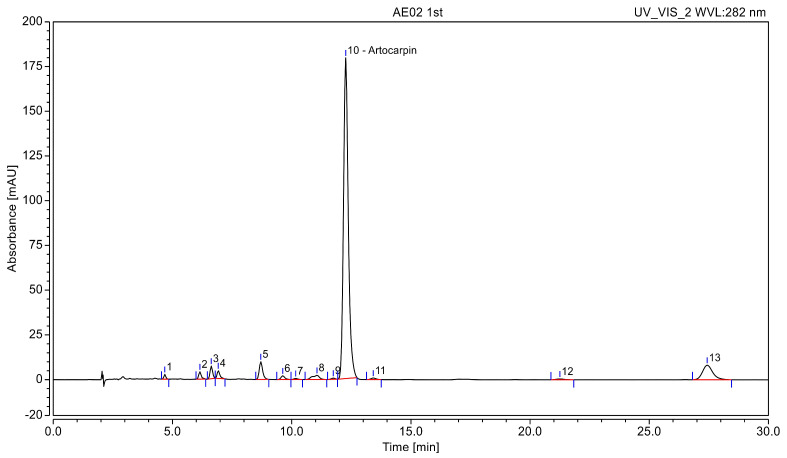
HPLC chromatogram of Artocarpin.

An HPLC method was developed for the simultaneous determination of artocarpin from Artocarpus heartwood extracts. The analysis was performed using an acetonitrile and water system with UV detection at 282 nm. Artocarpin, identified as peak 10, was the predominant compound in extract, eluting within 13 min (Figure 1). The amount of artocarpin in the extract was 0.35 ± 0.03 mg/mL according to HPLC. In addition, chromatographic peak area normalization revealed that artocarpin accounted for approximately 81% of the total peak area, indicating a high level of purity. Therefore, this extract can be regarded as a purified artocarpin-rich fraction.

### 3.2. Fabrication of Artocarpin Loaded Chitosan Microparticles

**Figure 2:**
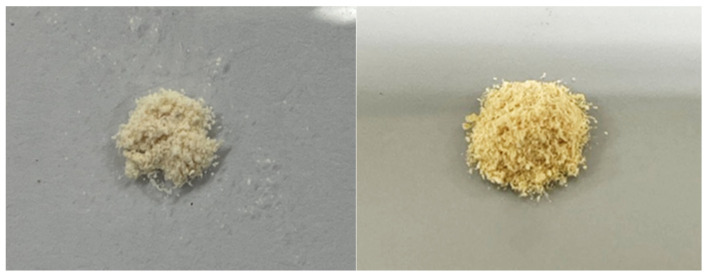
Product of chitosan microparticle (**left**) and artocarpin-loaded chitosan particles (**right**).

Particles of chitosan were produced based on the ion gelation method developed [21] by Calvo et al. [22] and then refined by Mulia et al. [18] as a result of the reaction between positively charged amino groups of chitosan and negatively charged TPP groups. Chitosan microparticles were developed using varying concentrations of chitosan and TPP solutions at different ratios. Distinct precipitation patterns were observed among the formulations, indicating the influence of component concentration in microparticle formation. The optimal formulation consisted of 0.1% chitosan solution and 0.5% TPP solution at a 5:1 volumetric ratio. Particle size analysis demonstrated that the use of 0.5% TPP led to a reduction in particle size, likely due to improved cross-linking efficiency and more compact polymer structures. Following optimization, artocarpin was incorporated into the optimal formulation at concentrations of 0.1, 0.5, and 1 µg/mL. Among these, the 0.5 µg/mL artocarpin formulation yielded microparticles with the most desirable physicochemical properties, including favorable particle size and morphology. Upon drying, the microparticles formed a fine powder, as illustrated in Figure 2.

Furthermore, incorporation of artocarpin at 0.5 µg/mL appeared to provide an optimal balance between drug loading and structural integrity. In contrast, a higher concentration (1 µg/mL) may have exceeded the encapsulation capacity, resulting in particle aggregation or reduced uniformity, while a lower concentration (0.1 µg/mL) may have led to insufficient drug loading and limited therapeutic potential. In addition, increasing the TPP concentration up to 0.5% or adjusting the chitosan:TPP ratio to 5:1 led to a noticeable reduction in particle size, likely due to enhanced ionic cross-linking that promoted a more compact and stable polymer matrix [23]. However, further increases in TPP concentration or lower chitosan:TPP ratios were associated with excessive cross-linking, which could induce particle aggregation and a broader size distribution [24].

### 3.3. Encapsulation Efficiency

**Figure 3:**
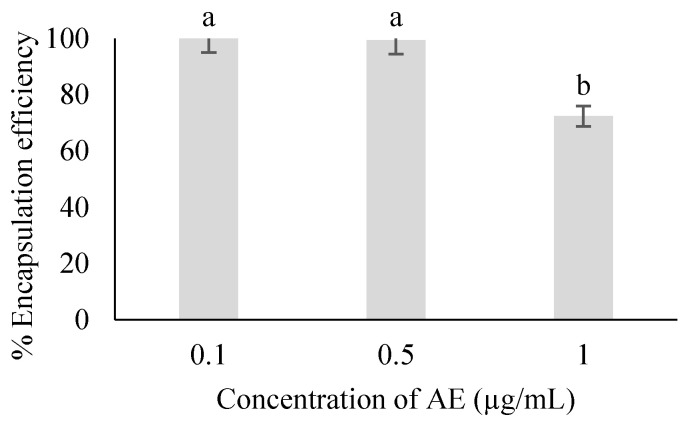
Encapsulation efficiency percentage of chitosan microparticles loading artocarpin. Different letters (a,b) indicate statistically significant differences among groups (*p* < 0.05).

The EE of microparticles loaded with artocarpin extract demonstrates the effectiveness of the optimized formulation in retaining bioactive compounds within the particles. Particles containing 0.1 µg/mL artocarpin exhibited the highest EE at 100%, followed by 99.46% for 0.5 µg/mL, and 72.43% for 1 µg/mL, as shown in Figure 3. These results suggest that lower concentrations allow for more effective interaction between artocarpin and the chitosan matrix, likely due to sufficient binding capacity and optimal electrostatic and hydrogen bonding interactions. In contrast, the decline in EE at 1 µg/mL may result from matrix saturation and limited availability of active binding sites, leading to unencapsulated residues and potential drug aggregation phenomena commonly observed in polymer-based delivery systems [25]. High EE is a desirable characteristic in particle drug delivery, as it enhances formulation efficiency, minimizes drug loss, and contributes to improved release control and therapeutic performance [22].

### 3.4. Cumulative Release

**Figure 4:**
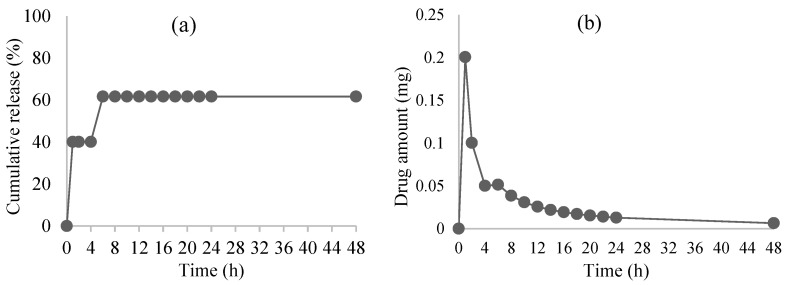
The in vitro release of the chitosan microparticles loading artocarpin (**a**). The drug release of the chitosan microparticles loading artocarpin (**b**).

The release profile of artocarpin from chitosan microparticles showed a clear biphasic pattern, with an initial burst release observed during the first 2–12 h, followed by a slower sustained release phase (Figure 4). The total release was approximately 60%, corresponding to about 0.23 mg released within the first 2 h. This release behavior suggests that the chitosan matrix can effectively facilitate an early rapid release, while maintaining a controlled and prolonged release of the hydrophobic bioactive compound. This biphasic behavior is commonly observed in polymer-based drug delivery systems and is attributed to the rapid diffusion of surface-associated drug during the initial phase and gradual diffusion and polymer degradation during the later phase [26]. The initial burst may facilitate prompt therapeutic action, while the sustained release prolongs drug availability, potentially reducing dosing frequency and enhancing patient compliance. The observed release kinetics suggest that electrostatic and hydrogen bonding interactions between chitosan and artocarpin, along with factors such as particle size and cross-linking density, contribute significantly to modulating the release rate [27].

### 3.5. Zeta Potential

Particle size and distribution of CSPs/AE exhibited a Z-average size of 3361 ± 151.99 nm with a polydispersity index (PdI) of 0.79 ± 0.18, as shown in Table 1. Compared to the particles without artocarpin, the chitosan particles encapsulated with artocarpin exhibited a higher positive zeta potential. The increased positive zeta potential observed after artocarpin encapsulation is likely due to its interaction with chitosan. Although artocarpin lacks a permanent positive charge, it does not suppress chitosan’s inherent positive charge and may enhance its surface expression by modifying particle surface characteristics. A positive zeta potential (25.00 mV) was observed in the study of Fahmy et al. [28]. Zeta potential of 31.1 mV from Bouzouita et al. [29] for green synthesis of chitosan nanoparticles optimized for multidrug-resistant biofilm-forming *Acinetobacter baumannii*. A higher zeta potential indicates stronger repulsive forces between particles, which helps prevent aggregation and supports prolonged dispersion stability. Therefore, the elevated zeta potential in CSPs/AE potentially contributes to enhanced particle suspension and stability during storage and biological application. A high PDI value would imply poor control over particle formation, leading to significant size variation, which may negatively impact drug release behavior, cellular uptake, and reproducibility. However, artocarpin does not reduce the positive charge of chitosan but rather enhances its expression, which may enhance its drug delivery potential or its adhesion to negatively charged target cells.

### 3.6. Accelerated Stability Studies

The stability evaluation of CSPs and CSPs/AE over 90 days at 4 ± 2 °C and 60 ± 5% RH demonstrated a gradual increase in particle size, indicating potential particle aggregation over time (Table 2). Specifically, CSPs increased from 2189.33 ± 84.18 nm to 4032.67 ± 156.25 nm, while CSPs/AE exhibited a larger initial size and reached 4535.33 ± 189.03 nm by day 90. The greater increase in particle size observed in CSPs/AE suggests that the incorporation of artocarpin may contribute to greater instability, potentially due to intermolecular interactions or disruption of the polymeric matrix. These findings are consistent with previous reports that chitosan-based particles may undergo aggregation or swelling during storage due to partial degradation or reduced electrostatic repulsion over time, particularly in systems lacking sufficient cross-linking or stabilization agents [30].

### 3.7. Characterization of Particles

#### 3.7.1. FTIR Analysis

**Figure 5:**
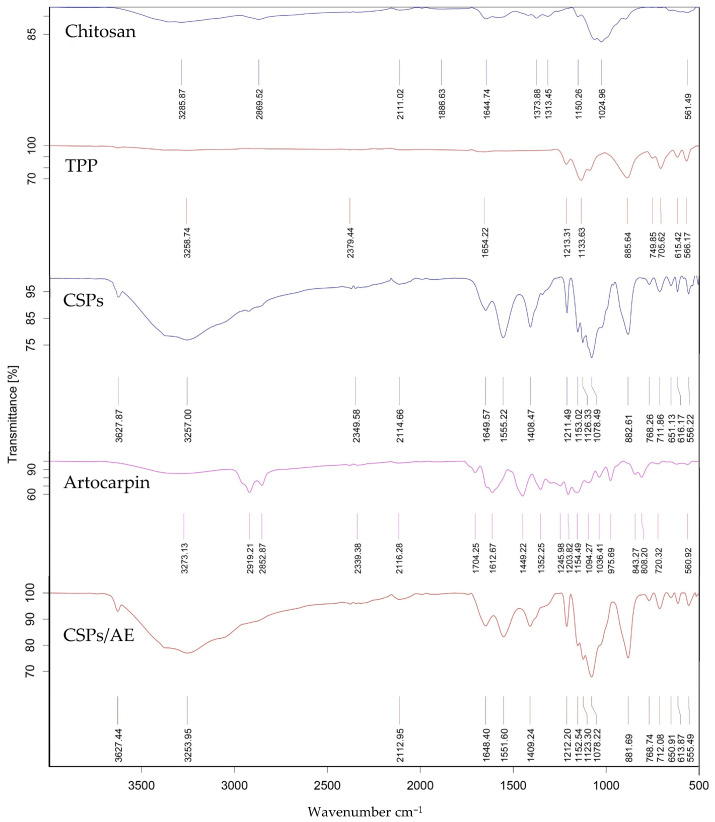
Spectrum of chitosan particles compared with artocarpin-loaded chitosan particles.

The interaction of artocarpin with the components of the chitosan-based particles was investigated using FTIR, as shown in Figure 5. This technique provided insights into the chemical bonding and molecular interactions that occur during particle formation and artocarpin encapsulation. The FTIR spectrum of CSPs/AE exhibited characteristic peaks, including a broad peak at 3627.44 cm^−1^ corresponding to O-H and N-H stretching, and peaks at 1648.40 cm^−1^ and 1551.60 cm^−1^ indicating C=O stretching and N-H groups, respectively. These findings are consistent with the known functional groups of chitosan, a polysaccharide with hydroxyl, amino, and carbonyl groups that contribute to its biocompatibility and cross-linking capabilities.

TPP, used as a cross-linking agent, showed prominent peaks at 1133.63 cm^−1^ and 885.64 cm^−1^, attributed to P=O and P-O-P stretching vibrations, respectively. These functional groups are essential for forming ionic cross-links with the amino groups of chitosan, stabilizing the particle structure. The FTIR spectrum of artocarpin revealed multiple peaks corresponding to its chemical composition. These included a peak at 2919.21 cm^−1^ for C-H stretching, 1704.25 cm^−1^ for C=O stretching, 1612.67 cm^−1^ for C=C stretching, 1449.22 cm^−1^ for C-H bending, and 1352.25 cm^−1^ for C-N stretching. These distinct peaks confirm the presence of flavonoid-specific functional groups, highlighting artocarpin’s potential bioactive properties. When comparing the FTIR spectra of artocarpin, the absence of peaks at 2919.21 and 2852.87 cm^−1^ (C-H stretching) in the loaded particles was noteworthy. This could indicate interactions between artocarpin and the chitosan matrix, potentially through hydrogen bonding or electrostatic interactions, which may alter the molecular environment and reduce or mask the infrared absorption at this wavenumber. Alternatively, the encapsulation process might cause the C-H stretching signal to diminish or disappear due to the integration of artocarpin into the particle structure, which alters its vibrational modes.

Similar FTIR spectral changes were observed in the chitosan-TPP system, where the appearance of phosphate-related peaks and slight shifts in amide bands indicated successful ionic cross-linking between TPP and the amino groups of chitosan, which is consistent with previous findings by Bhumkar and Pokharkar [31]. In CSPs/AE, the disappearance of C-H stretching peaks at 2919 and 2853 cm^−1^, normally present in free artocarpin, suggests that artocarpin was successfully incorporated into the chitosan matrix. This change may be due to hydrogen bonding or electrostatic interactions between artocarpin and the polymer network, which can reduce or mask specific vibrational signals. Similar results were reported by Zhang et al. [32], where drug-specific peaks were diminished after encapsulation, supporting the presence of molecular interactions within the delivery system.

#### 3.7.2. XRD Analysis

**Figure 6:**
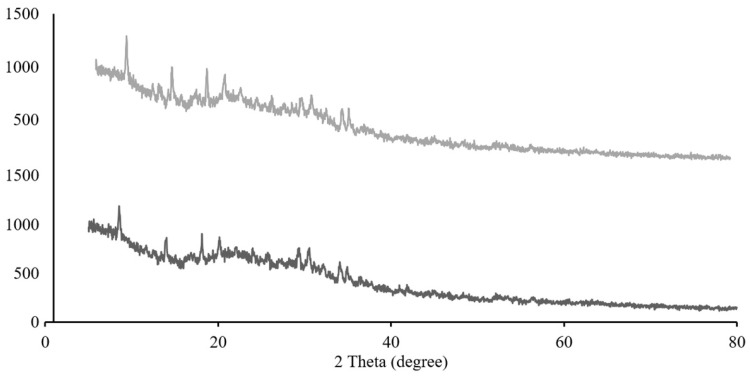
X-ray diffraction patterns of chitosan particles (top) and artocarpin-loaded chitosan particles (bottom).

XRD analysis (Figure 6) revealed that native chitosan particles exhibited characteristic semi-crystalline peaks at 2θ = 8.62°, 13.95°, and broad peaks from 15 to 35°, indicating both crystalline and amorphous regions typical of chitosan’s hydrogen-bonded polymer chains. These patterns align with previous reports showing peaks near 2θ = 10° and 20°, corresponding to the (020) and (110) planes of α-chitin [27].

Upon encapsulation of artocarpin, the diffraction pattern changed significantly. Artocarpin-loaded particles displayed peaks at 2θ = 8.52° and 18.11°, with broader peaks extending from 20 to 45°, suggesting disruption of chitosan crystallinity and a shift toward a more amorphous structure. This transformation may be due to interactions such as hydrogen bonding between chitosan and artocarpin, which can stabilize the encapsulated compound and alter interplanar spacing [33]. The reduction in crystallinity upon loading is a common feature in polymer-drug systems and often correlates with improved drug dispersion and controlled release. The emergence of new peaks in the loaded particles also indicates possible formation of new crystalline domains from the drug-polymer interaction [31].

#### 3.7.3. SEM Analysis

**Figure 7:**
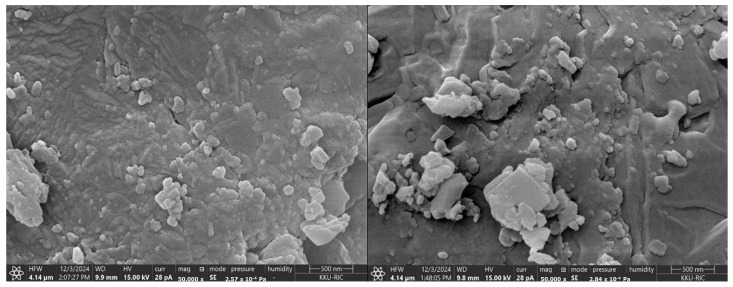
SEM micrographs showing the surface morphology of chitosan particles (**left**) and artocarpin-loaded chitosan particles (**right**). CSPs exhibited relatively smooth and compact structures, whereas CSPs/AE showed increased surface roughness and visibly adhered crystalline-like structures attributed to artocarpin loading. Images captured at 50,000× magnification with a scale bar of 500 nm.

The surface morphology and particle size analysis of CSPs prepared via polymerization provided valuable insights into their structural characteristics. SEM images (Figure 7) showed that both dried chitosan and artocarpin-loaded chitosan particles exhibited a distinct morphology, consisting of larger matrix particles with numerous smaller crystals of various shapes attached, indicating successful encapsulation of artocarpin within the chitosan polymer network. The predominantly square or nearly square shape of the larger particles suggests a controlled synthesis process yielding consistent particle formation. Such morphological uniformity is essential for ensuring predictable drug release profiles and pharmacokinetics [32]. The smaller crystals attached to the larger particles likely result from artocarpin precipitation during drying, influenced by interactions with the chitosan matrix.

#### 3.7.4. TEM Analysis

**Figure 8:**
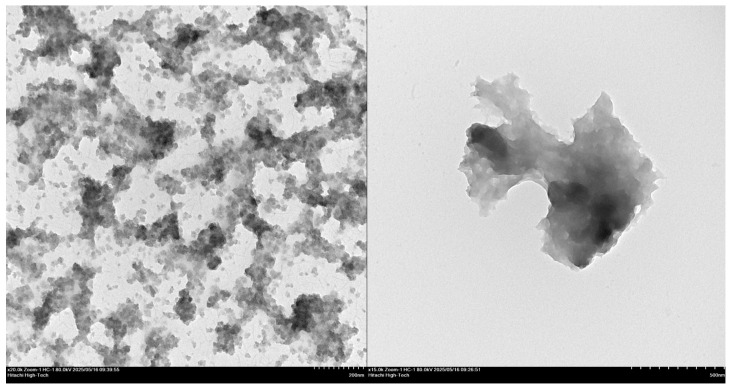
TEM images of chitosan particles (**left**) showing dispersed small structures and artocarpin-loaded chitosan particles (**right**) showing agglomerated masses with higher electron density. Scale bars: 200 nm and 500 nm.

Morphological characterization of the microparticles by TEM provided further insights into their structural properties. The TEM images (Figure 8) revealed microparticles with a predominantly spherical shape and size of approximately 1–2 µm. The images we observed closely resemble those depicted in reports found in the literature of Li et al. [34], Budi et al. [35], Guo et al. [36], and others. These morphological characteristics, such as the predominantly spherical shape and smooth surface, align with desirable features for biomedical applications. A spherical structure may help reduce nonspecific interactions with biological components and facilitate cellular uptake [34]. The smooth surface likely reflects a uniform coating of chitosan and TPP, which can contribute to colloidal stability and minimize aggregation. Moreover, the relatively uniform size distribution observed suggests a well-controlled synthesis process, which is important for ensuring consistent pharmacokinetic behavior and biodistribution in vivo.

### 3.8. Cytotoxicity Activity

**Figure 9:**
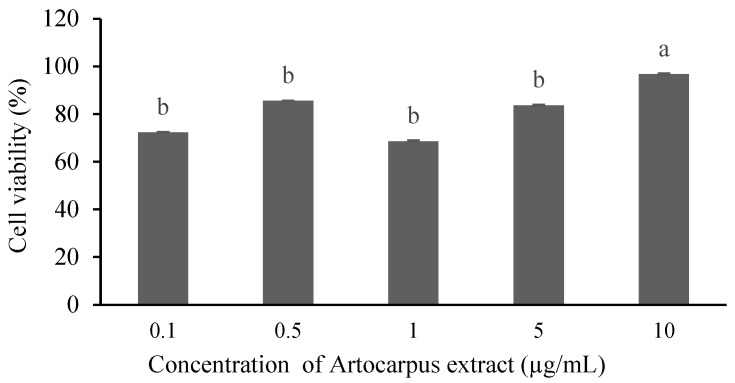
The RAW 264.7 cells viability of artocarpin (AE) at different concentrations. Different letter state significant different by Tukey’s test (*p* < 0.05).

The cytotoxicity assay is a critical preliminary step in evaluating the biosafety of plant-derived compounds, particularly when intended for biomedical applications such as drug delivery or anti-inflammatory therapies. Assessing cell viability allows us to identify safe concentration ranges that do not adversely affect normal cell function. In this study, the effect of Artocarpin on the viability of RAW 264.7 macrophage cells were investigated across a range of concentrations. The results demonstrated a concentration-dependent cytotoxicity pattern shoe in Figure 9. At lower concentrations (0.1–10 µg/mL), cell viability remained above 70%, with no statistically significant difference among these groups. This aligns with previous findings on the cytotoxic potential of polyphenolic or flavonoid-rich plant extracts when used at excessive doses [37].

**Figure 10:**
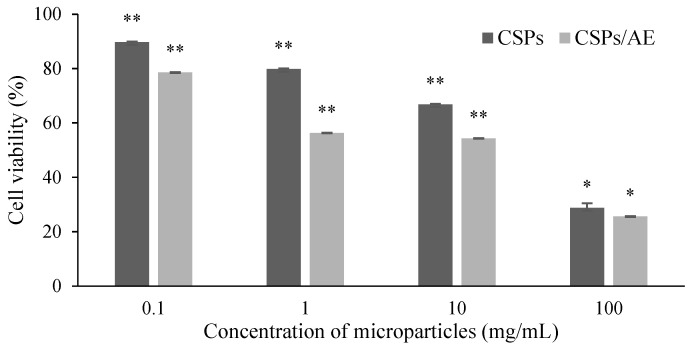
RAW 264.7 cell viability of chitosan microparticles (CSPs) and chitosan-loaded artocarpin (CSPs/AE) at different concentrations. Data are presented as mean  ±  SD. Asterisks indicate statistically significant differences compared with the untreated control group (* *p* < 0.05, ** *p* < 0.01). No significant differences were detected between CSPs and CSPs/AE at equivalent concentrations.

The cytotoxicity of the developed chitosan particles and artocarpin-loaded chitosan particles was evaluated using the MTT assay on RAW 264.7 cells, a widely used macrophage cell line for assessing biocompatibility and inflammatory responses. The assay tested a range of particle concentrations (0.1–100 mg/mL) to determine their effects on cell viability after 24 h of exposure. The results demonstrated that at lower concentrations of 0.1 and 1 mg/mL (Figure 10), both plain chitosan particles and artocarpin-loaded chitosan particles showed no observable cytotoxicity toward to RAW 264.7 cells, indicating excellent biocompatibility. Dose dependent cytotoxicity pattern is typical for many nanoparticle systems, as higher concentrations may lead to particle aggregation or excessive interactions with cellular membranes, causing reduced cell viability [37]. Furthermore, the encapsulation of artocarpin did not significantly alter the cytotoxic profile of the particles.

### 3.9. NO Production Assays

**Figure 11:**
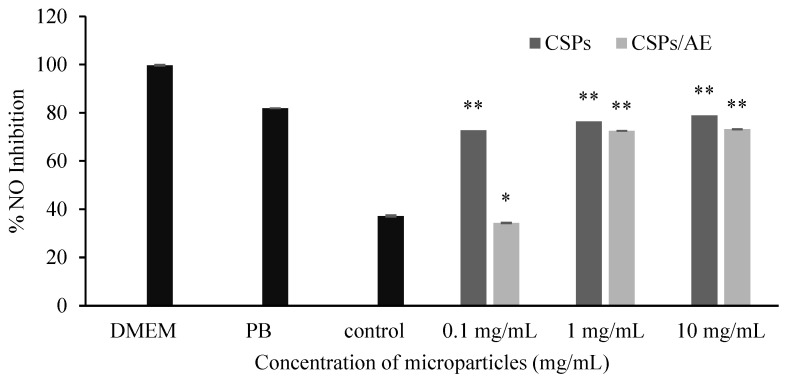
Nitric oxide (NO) inhibition in RAW 264.7 macrophages treated with chitosan particles (CSPs) and artocarpin-loaded chitosan particles (CSPs/AE) at different concentrations. Data are presented as mean  ±  SD. Asterisks indicate statistically significant differences compared with the untreated group (* *p* < 0.05; ** *p* < 0.01).

NO is an important inflammatory mediator that is produced by activated macrophages, mainly through the inducible Nitric Oxide Synthase (iNOS) pathway [38]. When produced in excess, NO can lead to oxidative stress, chronic inflammation, and tissue damage. Therefore, reducing NO production in macrophages is commonly used as an indicator of anti-inflammatory activity in cell-based studies. The results of %NO inhibition (Figure 11) show that both CSPs and CSPs/AE significantly reduced NO production in activated macrophages in a concentration-dependent manner (*p* < 0.05). CSPs inhibited NO production by 72.70%, 75.37%, and 75.86% at 0.1, 1, and 10 mg/mL, respectively, whereas CSPs/AE showed inhibition of 34.33%, 72.51%, and 73.19% at the same concentrations. At 0.1 mg/mL, NO inhibition by CSPs was significantly higher than CSPs/AE (*p* < 0.05), indicating a lower early release of artocarpin from the composite matrix. However, at 1 and 10 mg/mL, no significant differences were observed between CSPs and CSPs/AE (*p* > 0.05), suggesting that the release of artocarpin at these concentrations was sufficient to produce anti-inflammatory activity comparable to CSPs alone. Chitosan-based nanoparticles have been widely studied for their biocompatibility and anti-inflammatory properties, often attributed to their ability to modulate macrophage activity and reduce oxidative stress [39]. Artocarpin has been shown to exhibit anti-inflammatory properties, potentially by downregulating iNOS expression or interfering with signaling pathways involved in macrophage activation [40].

## 4. Conclusions

The optimized formulation for artocarpin delivery consisted of chitosan at 0.1% cross-linked with 0.5% sodium tripolyphosphate at a 5:1 ratio, which produced the smallest hydrodynamic diameter of approximately 3.3 µm and the highest encapsulation efficiency at a loading of 0.5 µg/mL. The particles exhibited an initial burst release between 1 and 12 h, followed by a sustained-release phase that reached a cumulative release of about 60%. Structural characterization using SEM, XRD, and FTIR confirmed the formation of spherical microparticles with an average size of approximately 16 µm in the dried state and verified the successful incorporation of artocarpin into the chitosan matrix. Biological evaluation demonstrated significant reductions in nitric oxide production at all tested concentrations, with CSPs showing inhibition values ranging from 72.70% to 75.86% and CSPs/AE achieving inhibition between 34.33% and 73.19%, with statistical significance at *p* < 0.05. Although the formulation showed promising anti-inflammatory potential, the study remains limited to in vitro assessment and does not include mechanistic validation or long-term stability data. Future research should focus on in vivo anti-inflammatory studies, pharmacokinetic profiling, and stability optimization to further establish the therapeutic applicability of artocarpin-loaded chitosan microparticles.

## Figures and Tables

**Table 1:** Zetasizer results show the average diameter size (nm) of the particles, zeta potential, and PdI.

Formulations	z-Avarage ± S.D. (d. nm)	Zeta Potential (mV)	PdI
CSPs	2733.33 ± 81.03	1.51	0.69 ± 0.15
CSPs/AE	3361 ± 151.99	12.8	0.79 ± 0.18

**Table 2:** Stability study conducted on the CSPs and CSPs/AE over 90 days at 4 ± 2 °C and 60 ± 5% RH.

Time (Days)	z-Avarage ± S.D. (d. nm)		Zeta Potential (mV)		PdI	
	CSPs	CSPs/AE	CSPs	CSPs/AE	CSPs	CSPs/AE
0	2189.33 ± 84.18	3527 ± 78.35	1.48	12.50	0.40 ± 0.84	0.58 ± 0.08
6	2616.67 ± 53.79	3769 ± 84.45	1.53	13.40	0.58 ± 0.85	0.57 ± 0.13
30	3787.33 ± 69.31	3856.33 ± 77.78	1.89	14.40	0.61 ± 0.11	0.79 ± 0.10
90	4032.67 ± 156.25	4535.33 ± 189.03	2.17	17.20	0.97 ± 0.04	0.92 ± 0.11

## Data Availability

The original contributions presented in the study are included in article; further inquiries can be directed to the corresponding authors.
